# Cost of a new method of active screening for human African trypanosomiasis in the Democratic Republic of the Congo

**DOI:** 10.1371/journal.pntd.0008832

**Published:** 2020-12-14

**Authors:** Rian Snijders, Alain Fukinsia, Yves Claeys, Alain Mpanya, Epco Hasker, Filip Meheus, Erick Miaka, Marleen Boelaert

**Affiliations:** 1 Department of Public Health, Institute of Tropical Medicine, Antwerp, Belgium; 2 Programme National de Lutte Contre la Trypanosomiase Humaine Africaine, Kinshasa, the Democratic Republic of Congo; 3 Section of Cancer Surveillance, International Agency for Research on Cancer, Lyon, France; Foundation for Innovative New Diagnostics (FIND), SWITZERLAND

## Abstract

**Background:**

Human African trypanosomiases caused by the *Trypanosoma brucei gambiense* parasite is a lethal disease targeted for eradication. One of the main disease control strategies is active case-finding through outreach campaigns. In 2014, a new method for active screening was developed with mini, motorcycle-based, teams. This study compares the cost of two active case-finding approaches, namely the traditional mobile teams and mini mobile teams, in the two health districts of the Democratic Republic of the Congo.

**Methods:**

The financial and economic costs of both approaches were estimated from a health care provider perspective. Cost and operational data were collected for 12 months for 1 traditional team and 3 mini teams. The cost per person screened and diagnosed was calculated and univariate sensitivity analysis was conducted to identify the main cost drivers.

**Results:**

During the study period in total 264,630 people were screened, and 23 HAT cases detected. The cost per person screened was lower for a mini team than for a traditional team in the study setting (US$1.86 versus US$2.08). A comparable result was found in a scenario analysis, assuming both teams would operate in a similar setting, with the cost per person screened by a mini team 15% lower than the cost per person screened by a traditional team (1.86 $ vs 2.14$). The main explanations for this lower cost are that mini teams work with fewer human resources, cheaper means of transportation and do not perform the Capillary Tube Centrifugation test or card agglutination test dilutions.

**Discussion:**

Active HAT screening with mini mobile teams has a lower cost and could be a cost-effective alternative for active case-finding. Further research is needed to determine if mini mobile teams have similar or better yields than traditional mobile teams in terms of detections and cases successfully treated.

## Author summary

Human African Trypanosomiasis (HAT) used to be a major public health problem in Sub-Saharan Africa, but the disease is becoming less frequent as a result of sustained control efforts. Currently, the elimination of sleeping sickness as a public health problem is targeted for 2020 and eradication or interruption of transmission for 2030. To achieve these targets, a commitment of at least 10 years towards HAT control activities will be necessary with innovative disease control approaches accompanied by economic evaluations to assess their cost and cost-effectiveness in the changing context. Today, active case finding via mass outreach campaigns accounts for approximately half of all identified cases in the Democratic Republic of the Congo. However, this strategy has become less efficient, with a dwindling “yield” in terms of the number of identified cases, translating to a higher cost per diagnosed HAT case. Therefore, different approaches to outreach campaigns need to be evaluated with a focus on reaching populations at risk for HAT.

This article presents the costs and outcomes of two approaches to active screening: traditional mobile teams and mini mobile teams.

This study shows that mini mobile teams could be a cost-effective alternative for active screening with a cost-per-person screened of US$1.86 compared to US$2.08. Improved efficiency could increase the screening coverage of populations at risk for HAT that are currently not being reached through the traditional approach. Future research is needed to evaluate the difference in HAT cases identified and treated by both approaches.

## Introduction

Human African trypanosomiasis (HAT), or sleeping sickness, is a vector-borne disease believed to be invariably fatal when left untreated. There are two forms of HAT, one caused by the Trypanosoma brucei (*T*. *b*.*) rhodesiense* and a second caused by the parasite *T*. *b*. *gambiense*. Infections with *T*.*b*. *gambiense* are responsible for more than 95% of the globally reported HAT cases and are the focus of this study.[1]

HAT is considered a public health problem because of the devastating epidemics in the 20^th^ century, but it is becoming more and more uncommon today thanks to sustained control efforts.[2] Therefore, the World Health Organization’s (WHO) Strategic and Technical Advisory Group for neglected tropical diseases decided to target the elimination of HAT as a public health problem by 2020 (annually i. less than 2,000 cases globally and ii. < 1 case/10,000 people in areas at moderate or higher risk) and interruption of transmission by 2030.[3] In 2019, 864 new HAT cases were declared globally, well below the targeted maximum of 2,000 cases.[4]

The current method to control HAT is a combination of case-finding and treatment, and in some places, vector control as well. Case-finding in the DRC is conducted either actively, through mass outreach campaigns by large 7 to 9 people, truck based mobile teams (here after called ‘traditional teams’) or passively in fixed health facilities. Currently, each of these strategies’ accounts for approximately half of all identified cases. Active case-finding has proven to be highly effective in poor, remote HAT endemic communities with limited access to health care facilities, but this strategy is labour-intensive, costly, and time-consuming as it generates a high opportunity cost for the populations screened due to the time they have to queue waiting for the service.[5,6] In a context of near disease elimination, this control strategy also becomes less efficient, with a dwindling “yield” in the number of identified HAT cases, translating to a higher cost per detected case due to the decreasing prevalence and declining participation rates. Additionally, mass screening campaigns are characterised by heavy logistics operations which limit the possibilities of targeted and responsive screening in high-problem areas and in remote areas that are difficult to access by car.[7]

Five years ago, an alternative model for active HAT screening by “mini-teams”, 4 people with motorcycles, was developed. which tries to mitigate the diminishing uptake and efficiency of traditional teams.[8] Qualitative research showed that communities prefer this type of screening because it is more adapted to their daily routine and guarantees more confidentiality, and therefore, they are also more likely to participate.[7,9]

Despite the importance of acceptability, rational use of resources and operational issues, past estimates of costs are outdated and innovative methods currently available were not considered. Only a few economic evaluations assess the cost and cost-effectiveness of HAT control activities, and they mainly focus on diagnostic algorithms for case detection, treatment options, and vector control.[6] If we want to achieve sustainable elimination of transmission, a commitment towards HAT control activities will be necessary, integrating improved tools and innovative disease control approaches.[10] This study documents the cost of two approaches to active HAT screening: traditional mobile teams and mini mobile teams, aiming to facilitate decisions on resource allocation for HAT control in different settings in the context of disease elimination.

## Materials and methods

### Ethics approval

Ethical approval for this study was obtained from the Institutional Review Board (IRB) of the Institute of Tropical Medicine, Belgium, (IRB/AB/ac/137 –Protocol number 115/16) as well as from the IRB at Ecole de santé Publique of the University of Kinshasa, RDC (ESP/CE/08/2017).

The study did not make use of human or animal subjects and/or tissue as it evaluated costs and aggregated operational data of routine activities provided by the health care system. Therefore no formal consent was needed.

### Study area & study period

**Fig 1:**
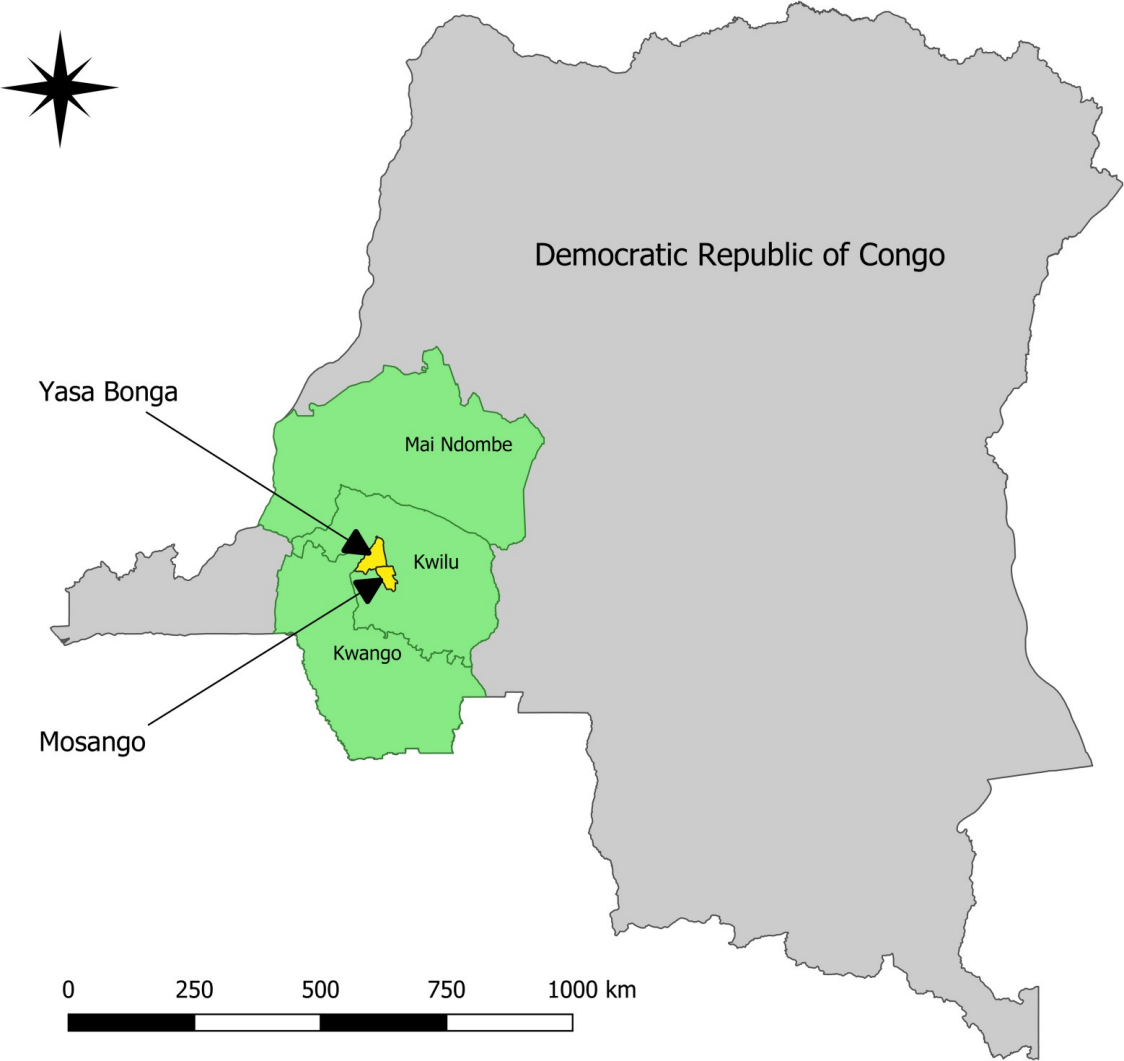
Location of the study area (map generated using QGIS 3.6.1)[11].

The study was conducted from May 2017 to April 2018 in two rural high-prevalence health zones in the Kwilu province: Mosango and Yasa Bonga (Fig 1) Active & passive screening for HAT has been conducted for many years in this region with the introduction of mini mobile teams in both zones and vector control in Yasa Bonga since 2016.

### Mobile screening strategies and diagnostic algorithms in DRC

A traditional mobile team consists of 8 to 9-members that travel from village to village using a truck. These teams invite the whole community to a centrally located open space in the village. A “mini-team” consists of only 4 people; three members of the team perform screening by visiting every family in a community on a door-to-door calling basis and the fourth team member performs parasitological confirmation of HAT suspects at a later moment. Both the traditional mobile team and mini team screen on average 300 people per day for 20 days a month for 11 months a year. The screening capacity of both types of teams is therefore estimated at 66,000 people annually. The organisational differences are summarized in Table 1.

**Table 1:** Organisational differences.

	Traditional Mobile Team	Mini Mobile Team
Human Resources	8 people	4 people
Vehicle	One 4x4 vehicle	4 motorcycles
Energy source	Diesel generator & batteries	4 solar panels & batteries
Screening period	12 months	12 months
Average daily screening target	300 (20 days/month)	300 (20 days/month)

#### Diagnostic algorithms

HAT diagnosis needs to be confirmed before a patient can be treated because of the toxicity and complexity of HAT treatment regimes. The disease is currently diagnosed through a combination of a serological test followed by more specific parasitological tests. The most frequently used serological test is the Card Agglutination Test for Trypanosomiasis (CATT), which is appropriate for mass population screening and distributed in vials of 50 tests. Once opened, the vials need to be used the same day, and specialised equipment (a rotator) requiring electricity and a cold chain for storage is needed.[12,13] During the study period, both the traditional teams and mini teams performed the CATT test for screening. In July 2018 the mini teams started using rapid diagnostic tests (RDTs) for screening.

During the screening campaigns, the cervical lymph nodes of all people with a positive CATT test and/or typical HAT symptoms (HAT suspects) were palpated. Upon detection of typically swollen lymph nodes, a lymph gland puncture (LGP) was performed and the fluid examined for parasites. HAT suspects without typical lymph nodes or with a negative lymph node examination were referred for microscopy tests in the following sequence: Capillary Tube Centrifugation (CTC) followed by the more sensitive Mini Anion Exchange Centrifugation Technique (mAECT).[14,15] A HAT case was considered confirmed when one of the microscopy tests was positive (LGP, CTC, or mAECT). While traditional teams followed the national sleeping sickness program (Programme National de Lutte contre la THA or PNLTHA) guidelines, mini mobile teams did not perform the CTC since their main energy source is 12-volt batteries charged through solar panels. No suitable 12-volt haematocrit centrifuges necessary for CTC could be found. Furthermore, 2 centrifuges would be too cumbersome on a motorcycle.[14]

#### Disease staging, monitoring, and treatment

HAT evolves in two stages: a haemo-lymphatic stage followed by a meningo-encephalitic stage when the parasite penetrates the blood-brain barrier and affects the central nervous system. During the study, the WHO guidelines stated a different treatment for each stage, requiring staging of disease through a lumbar puncture (LP) once the presence of the parasite was confirmed in the blood.[1,16] First-stage HAT cases were treated with pentamidine and second-stage patients with nifurtimox-eflornithine combination therapy (NECT).[5]

Traditional teams performed the LP on the spot and usually carry pentamidine for stage-one treatment at the nearest health centre. Stage-two patients were referred to the nearest hospital because NECT treatment requires intravenous infusions and close clinical monitoring. Contrary to traditional teams, mini teams are not equipped for staging. Therefore, mini teams refer all confirmed HAT cases to hospitals for staging and treatment. In addition, traditional teams performed serial dilutions of CATT on HAT suspects with negative microscopy tests. People testing positive on CATT 1/8 are considered ‘serological cases’, to be staged and treated for HAT, like cases detected through LGP, CTC, or mAECT. People testing negative on CATT 1/8 were referred for monitoring by the local health centre.

**Fig 2:**
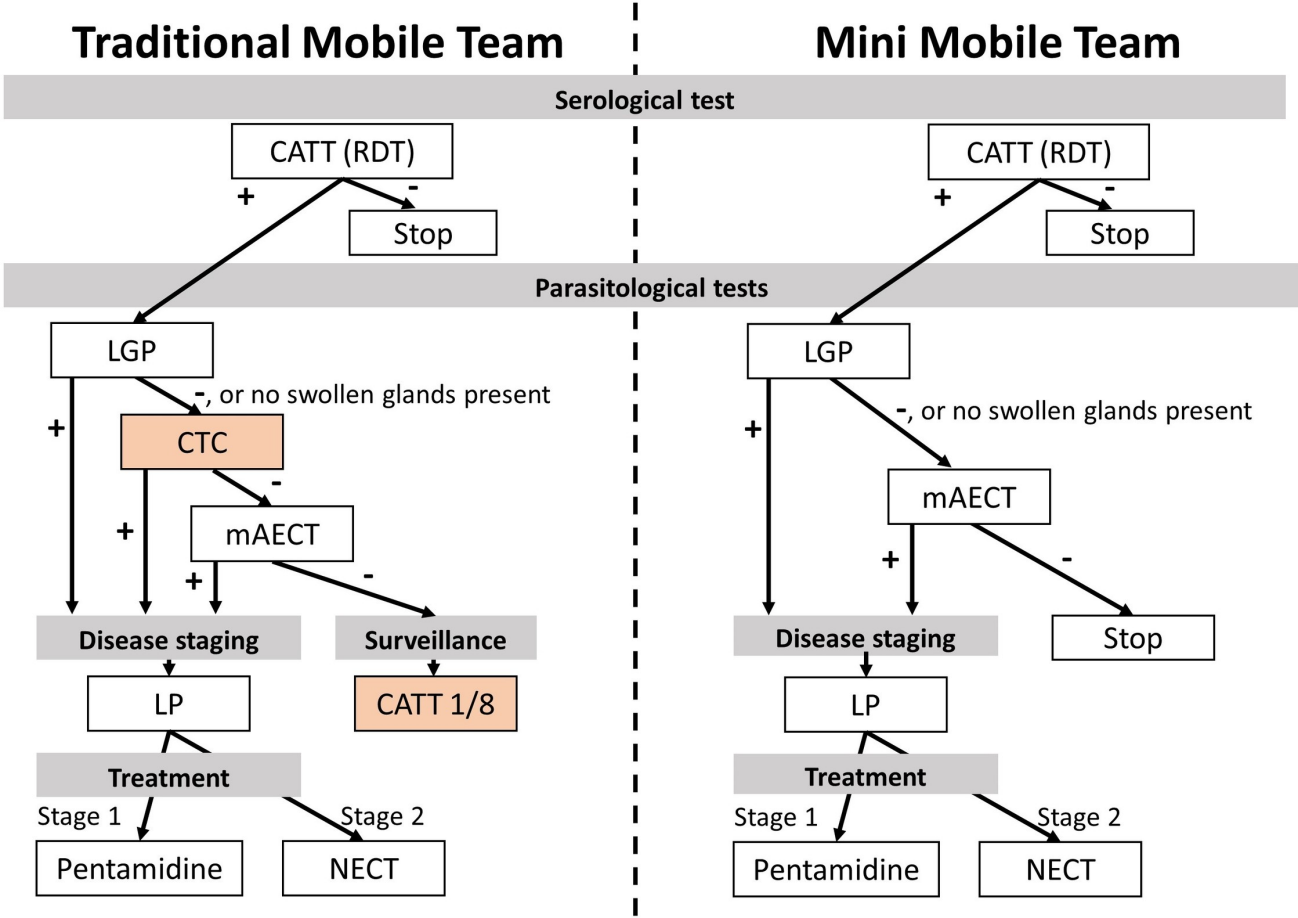
Differences in diagnostic algorithm.

Table 2 and Fig 2 provides an overview of the difference in screening staging, monitoring, and treatment.

**Table 2:** Difference in stage determination and treatment.

	Traditional Mobile Team	Mini Mobile Team
Stage determination	LP on site	LP in Health structure equipped to perform HAT microscopy & 2^nd^ stage treatment
Treatment stage 1	Nearby health centre	Nearby health centre
Treatment stage 2	Health structure equipped to perform HAT microscopy & 2^nd^ stage treatment	Health structure equipped to perform HAT microscopy & 2^nd^ stage treatment

### Costing methodology

The study adopted a health care provider perspective. Data on resource consumption and prices were collected prospectively and complemented with financial records from the HAT control programme. Costs incurred by households were excluded. Research costs for implementation were expressed in equivalent local costs.

Costs were categorised as recurrent or capital (defined as equipment with a useful life of more than one year). Both financial and economic costs were estimated. Financial costs represent the actual quantities consumed and prices paid for consumables, including transportation costs during the study period as well as any durable equipment that was purchased specifically for these activities. Economic costs were estimated as the value of resources foregone that could have been used in other activities (“opportunity costs”). For capital equipment, the purchase or replacement value was considered and annualised based on the expected useful life and discounted at a discount rate of 3%.[17]

For each approach, the annual cost was divided by the number of people screened for 12 months to calculate the annual cost per person screened. All costs exclude value-added taxes (VAT) since the PNLTHA and its main donors are VAT-exempt which is normally in the DRC. [18] For items shipped to the DRC, the price was increased by 10% based on the average shipment cost of goods between Europe and the DRC. All costs were recorded in the currency they were incurred in and converted to US$ following the average exchange rate of the study period (EUR to dollar: 1,18; CDF to dollar: 0,00065).

### Scenario analysis: Adjusting for differences in contexts

Because the data are incompatible with a traditional econometric analysis to control for epidemiological differences, we performed a small simulation study of the costs incurred by each team if they were to operate in populations that are epidemiologically identical.

In our modelled scenario, we assumed that both models have a similar percentage of serological suspects but that each team uses their regular diagnostic algorithm (Traditional team: LGP, CTC, mAECT, CATT 1/8; Mini team: LGP, CTC, mAECT).

Additionally, one-way sensitivity analysis was performed to consider the specific contribution, after control for background epidemiology, of the specificity and use of serological screening tests, the prevalence of the disease, performance of the mobile teams, impact of changes in the useful life of vehicles and motorcycles (maximum and minimum according to WHO Choice guidelines in Africa), discount rate, other important cost drivers such as the fuel cost and the price of mAECT, and the use of RDT’s as serological test. The diagnostic test and epidemiological parameters used for this scenario and the sensitivity analysis can be found in the supplementary information (S4 Table).

## Results

### Operational results

Between May 2017 and April 2018, the traditional team screened 65,190 people, performed microscopy tests on all 276 HAT suspects identified, and diagnosed 11 new HAT cases. The mini teams screened on average 66,480 people per team, performed microscopy tests on 95% of 833 HAT suspects, and diagnosed 6 new HAT cases (Table 3). The percentage of positive CATT tests was higher for the mini teams compared to the traditional teams (1.4% vs. 0.4%) leading to more parasitological confirmation tests that had to be performed by the mini teams. None of the CATT 1/8 tests performed by the traditional team on serological suspects were positive.

**Table 3:** Number of tests performed, and HAT cases identified between May 2017 and April 2018.

Type of team	CATT	CATT +	LNA	CTC	mAECT	HAT stage 1	HAT stage 2
Traditional Mobile Team	65,190	276 (0,42%)	17	220	271	4	7
Mini Mobile Team 1	67,715	839	242	-	811	1	1
Mini Mobile Team 2	61,958	806	60	-	772	5	10
Mini Mobile Team 3	69,767	855	108	-	801		
Average Mini Mobile Teams	66,480	833 (1.25%)	137	-	795	2	4
**Average all Mobile Teams**	**65,835**	**555**	**77**	**110**	**533**	**3**	**5**
**Total all Mobile teams**	**264,630**	**2,776 (1.05%)**	**427**	**220**	**2,655**	**10**	**18**

### Financial and economic costs

#### Financial costs

The average annual financial cost for a traditional team for a 10-year period was estimated at 158,189$ (between 125,733 and 228,191) and for a mini team around 124,640 $ (between 101,412 and 171,926). The annual variations can be explained by the expenses related to the replacement of capital equipment. (S1 Table).

#### Economic costs

Table 4 shows the economic costs per item for both approaches, the overall annual and the cost per person screened/diagnosed. The cost per person screened by a mini team (1.86$) was 12% lower than for a traditional team (2.08$) while the cost per person diagnosed is 44% lower for a traditional team (12,302$) compared to a mini team (21,893$) due to the higher number of cases detected by the traditional team. Detailed information per cost item is available in S2 Table.

**Table 4:** Annual economic costs of active screening in Yasa Bonga and Mosango, by screening approach.

	Traditional Team		Mini team	
	Cost (US$)	%	Cost (US$)	%
**Capital costs**	**10,782**	**8.0**	**8,582**	**6.9**
Vehicle/motorcycles	5,073	**3.7**	4,136	**3.3**
Medical and laboratory equipment	1,944	**1.4**	1,147	**0.9**
Energy source	344	**0.3**	419	**0.3**
Electronics	624	**0.5**	554	**0.4**
Other equipment	973	**0.7**	504	**0.4**
Training	1,823	**1.3**	1,823	**1.5**
**Recurrent costs**	**124,540**	**92.0**	**114,999**	**93.1**
Human Resources	31,074	**23.0**	20,892	**16.9**
Laboratory & medical supplies				
Screening tests	53,587	**39.6**	58,355	**47.2**
Parasitological confirmation	1,793	**1.3**	4,329	**3.5**
Staging	208	**0.2**	0	**0.0**
Surveillance	932	**0.7**	0	**0.0**
Other supplies and materials	5,615	**4.1**	5,045	**4.1**
Fuel costs	5,719	**4.2**	2,299	**1.9**
Management	25,611	**18.9**	24,080	**19.5**
**Total screening cost**	**135,322**	**100**	**123,582**	**100**
Cost per person screened	2.08		1.86	
Cost per person diagnosed with HAT	12,302		21,893	

### Scenario analysis: Costs in identical settings

Table 5 shows the cost per person screened for a theoretical scenario in which both types of teams operate in an identical context: 2.14$ for a traditional team and 1.86$ for a mini team. This cost is slightly higher for the traditional team than observed in Yasa Bonga and Mosango. In the model, the number of CATT positives is estimated based on the average number of CATT positives detected during the study (1.05%). The mini teams observed around 3 times more CATT positive tests than the traditional teams. This results in a higher number of CATT positives in the model for the traditional team and therefore a higher cost for parasitological confirmation and surveillance tests than observed in the study setting.

The overall cost per person screened by a mini team is 0.29$ or 15% lower than by a traditional team. Over 80% of this difference can be explained by the lower costs for human resources, means of transportation (motorcycles and fuel consumption) and difference in screening algorithm (no CTC or CATT dilution) of a mini team. This lower cost is partially offset by the higher screening test cost due to the higher CATT wastage (S3 Table).

**Table 5:** Results scenario analysis: Costs of a traditional and mini team in an identical setting.

	**Traditional Team**		**Mini team**		**Difference Cost per person screened**	
	$	%	$	%	$	%
**Capital equipment**	**10,782**	**7.6**	**8,582**	**7.0**	**0.03**	**11.6**
**Annual recurrent costs**	**130,751**	**92.4**	**114,039**	**93.0**	**0.25**	**88.4**
Human resources	31,074	22.0	20,892	17.0	0.15	53.8
Laboratory & medical supplies	61,922	44.8	61,849	50.4	0.00	0.4
Screening tests	54,253	38.3	57,934	47.2	-0.06	-19.5
Parasitological confirmation	5,140	3.6	3,915	3.2	0.02	6.5
Staging	114	0.1	-	0.0	0.00	0.6
Surveillance	2,415	1.7	-	0.0	0.04	12.8
Other supplies and materials	5,615	4.0	5,045	4.1	0.01	3.0
Fuel costs	5,719	4.0	2,299	1.9	0.05	18.1
Management	26,421	18.7	23,955	19.5	0.04	13.0
**Total screening**	**141,533**	**100**	**122,621**	**100**		
Cost per person screened	2.14		1.86		0.29	15.4
Cost per person diagnosed with HAT	23,551		22,888			

### One-way sensitivity analysis of cost drivers

**Fig 3:**
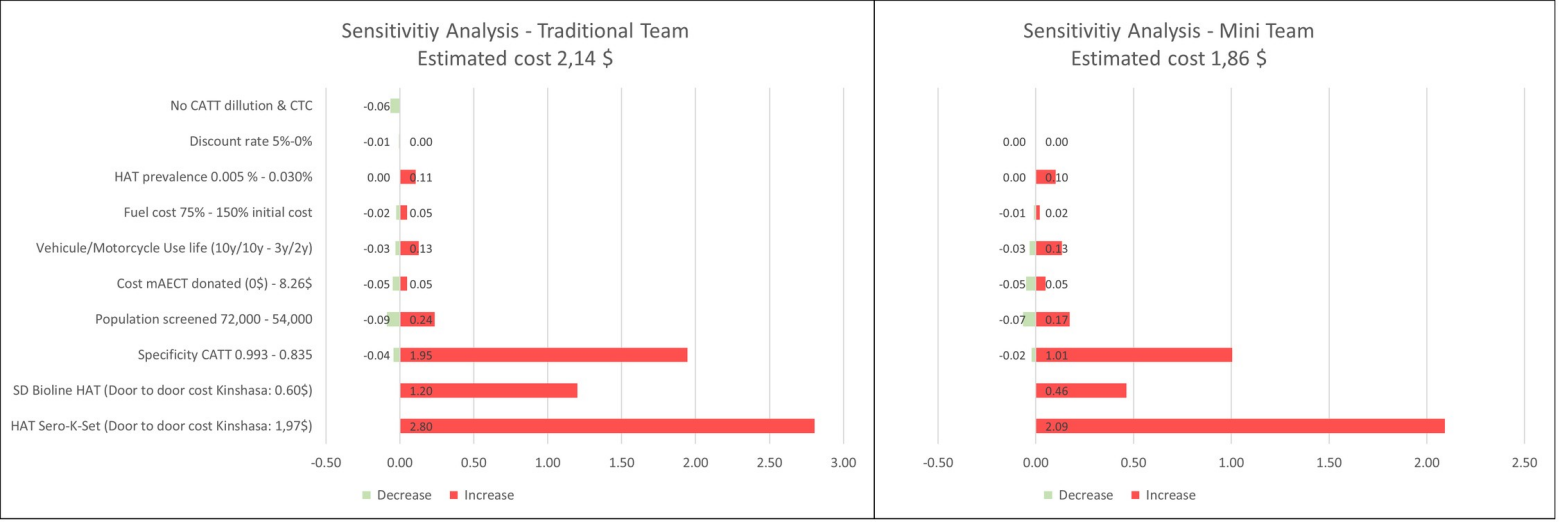
One-way sensitivity analysis–Additional cost/saving per person screened.

Fig 3 shows the impact on the cost per person screened when changing the variables used individually for the modelled scenario. (S4 Table)

For both approaches, the main cost drivers were the unit cost and the specificity of the serological test. Considering that the CATT has a high specificity (0.993) has a small impact on the cost per person screened because of the high number of cases detected among the CATT positive tests in the study area (28/2,776).[19] In a context of disease elimination with very low prevalence, a lower specificity of the serological test will result in a higher number of false positives and therefore more microscopy tests. Using serological tests with a higher or lower specificity has a big impact on the cost per person screened.

Since the beginning of 2018, the mini mobile teams use HAT RDTs for active screening. The SD Bioline HAT RDT (0.60$) is less expensive than the CATT (0.74 $) but includes a subsidy of 0.25$ per RDT produced paid externally to the supplier.[20] The HAT Sero-K-Set RDT (1.97$) is much more expensive. Additionally, the literature reports a lower specificity for both RDT’s than for the CATT which would result in more false positive serological tests and more microscopy tests needed. Therefore, the use of both RDT’s will push the cost per person screened upwards. When using the more expensive RDT (HAT Sero-K-Set) the cost per person screened could almost double for both approaches (4.95$ VS 2.14$; 3.95$ VS1.86$).[21]

During the study period, the diagnostic algorithm of a traditional team included 2 tests (CTC and CATT 1/8) that were not performed by the mini team. Excluding these tests from the traditional team’s diagnostic algorithm would lower their cost per person by 0.06$ to 2.08$ per person screened.

The study considered the purchase price of the mAECT, but currently, the gel needed to produce this test is donated. This sensitivity analysis also looked at the impact if this gel would no longer be donated. The cost per person screened would increase and the impact depends on the specificity of the serological tests.

The remaining variables (HAT prevalence, fuel cost, useful life of vehicles and motorcycles) have a much smaller impact on the cost per person screened.

## Discussion

The current paper is a contribution to cost HAT efforts in the DRC. This study demonstrates that the new active screening approach by mini teams costs less than screening with traditional teams. The results are valid in the study area (1.86 $ vs 2.08 $) as well as for a scenario analysis assuming both approaches are implemented in an identical setting (1.86 $ vs 2.14$). A costing study in 2007 reported a higher cost per person screened by a traditional team in context with a higher prevalence, namely between 1.96 € and 2,99 € (or 2.7 $ and 4.1 $ in 2018).[14] A cost-effectiveness analysis comparing the societal costs (including the costs for the patient) and outcomes for active screening by traditional mobile teams using CATT or RDT estimated the cost per person screened with CATT at 2.31 $ (VS 2.14$) and with RDT at 2.37 $ (VS 3.34$). The differences can mainly be explained by the inclusion of the patient costs and the differences in parasitological confirmation tests due to the use of a much lower specificity of the CATT and a higher specificity for the SD Bioline HAT in this model.[19]

Research showed that people at risk for HAT are more likely to participate in screening activities by a mini team as they are contacted in person, do not need to queue, the moment of screening can be adapted to their daily routines and their privacy is respected which could lead to a lower cost for the people screened.[9] Additionally, mini teams could reach areas inaccessible by vehicle, and investment and fuel costs of mini teams are much lower than for a traditional team, making them more suitable to be deployed in remote areas, regions inaccessible by car or to boost active screening activities for a short period.

A disadvantage in the current set up of mini teams is that they have difficulties ensuring that all HAT suspects undergo parasitological confirmation because such confirmation usually takes place at least 1 or 2 weeks later. Traditional teams, on the other hand, perform screening and confirmation simultaneously, ensuring continuity of care. The delay between screening and confirmation for mini teams could be resolved by making the screeners and the microscopist move around together but this adaptation of the strategy could mean that additional staff and equipment is needed which would increase the cost per person screened. In August 2018, WHO published new guidelines for the treatment of sleeping sickness following the approval of the oral medicine fexinidazole, which is used for both stages.[22] The problem would also be resolved if Fexinidazole which treats both stages or Acoziborole, a one dose drug against both stages and currently in the clinical trial phase, would be safe enough to treat serological HAT suspects making routine parasitological confirmation obsolete.[23] Additionally, mini teams and traditional teams use a different diagnostic algorithm (use of CTC and CATT dilution). It would make sense to stop using both additional tests in low prevalence contexts as they do not increase the sensitivity of the diagnostic algorithm yet increase the cost per person screened significantly.[24]

Currently, the mini mobile teams are using HAT RDTs, therefore their cost for active screening is most likely between 25% or even 115% higher than reported in this study, depending on the RDT they are using due to the higher purchase cost of the serological tests and the lower specificity. Additionally, active screening with filter papers is being reconsidered for active screening in low prevalence areas. Up to date cost data should be collected and this option should be included in future cost effectiveness studies regarding HAT outreach campaigns if this strategy would be implemented on a larger scale. [25–26]

Overall HAT screening by mini teams could be a cost-efficient alternative for active screening if they have similar or better outcomes in terms of the detection rate and enrolment in treatment. Better accessibility to populations at risk, the sensitivity of the diagnostic algorithm, and the delay between serological and parasitological tests could affect the number of cases identified and the enrolment in treatment and therefore the effectiveness of the teams. This approach should be considered a valid alternative to the traditional way of active HAT screening, but further research is needed to evaluate the difference in HAT cases identified and treated. This would allow comparing the outcomes of both strategies and evaluating the cost-effectiveness in terms of cost per person diagnosed and treated.

## Supporting information

S1 Table Annual financial cost. (PDF)

S2 Table Economic costs. (PDF)

S3 Table Scenario analysis: Baseline scenario with both teams operational in a similar setting. (PDF)

S4 Table Sensitivity analysis. (PDF)
