# Supplementary material for: Cost of a new method of active screening for human African trypanosomiasis in the Democratic Republic of the Congo
Source: PLoS Negl Trop Dis. 2020 Dec 14;14(12):e0008832. doi: 10.1371/journal.pntd.0008832 (PMC7769601; doi:10.1371/journal.pntd.0008832)
Supplement: S1 Table — (PDF) [file pntd.0008832.s001.pdf]

S1 Annual financial cost

Table 1 Assumptions financial costs

**Assumptions**	
People screened	66,000
Prevalence	0.013%
Sensitivity CATT	0.953
Specificity CATT	0.948
Sensitivity algorithm LNA-CTC-mAECT	0.903
Sensitivity algorithm LNA-mAECT	0.805
% of CATT discarded Traditional Team	7.5%
% of CATT discarded Mini Team	15%
Number of cases	9
Number of confirmations	3,440
Number of cases identified by traditional team (LNA_CTC_mAECT)	7.4
Number of cases identified mini team (LNA-mAECT)	6.6

Table 2 Financial costs by year (undiscounted)

	Year 1	Year 2	Year 3	Year 4	Year 5	Year 6	Year 7	Year 8	Year 9	Year 10			
	**Traditional Team**												
**Costs at the mobile team level**													
**Capital Equipment**	**56,052 $**	**0 $**	**7,779 $**	**280 $**	**9,616 $**	**7,417 $**	**8,059 $**	**38,740 $**	**9,616 $**	**280 $**			
Vehicle/motorcycles	38,740 $	0 $	0 $	0 $	0 $	0 $	0 $	38,740 $	0 $	0 $			
Medical and laboratory equipment	5,181 $	0 $	3,179 $	0 $	3,179 $	2,001 $	3,179 $	0 $	3,179 $	0 $			
Energy source	1,821 $	0 $	0 $	0 $	0 $	1,821 $	0 $	0 $	0 $	0 $			
Electronics	3,595 $	0 $	0 $	0 $	0 $	3,595 $	0 $	0 $	0 $	0 $			
Other equipment	3,016 $	0 $	900 $	280 $	2,736 $	0 $	1,180 $	0 $	2,736 $	280 $			
Training	3,700 $	0 $	3,700 $	0 $	3,700 $	0 $	3,700 $	0 $	3,700 $	0 $			
**Annual Recurrent costs**	**172,139 $**	**163,731 $**	**164,898 $**	**163,773 $**	**165,174 $**	**164,844 $**	**164,940 $**	**169,542 $**	**165,174 $**	**163,773 $**			
Lab & medical supplies - Screening tests	54,253 $	54,253 $	54,253 $	54,253 $	54,253 $	54,253 $	54,253 $	54,253 $	54,253 $	54,253 $			
Lab & medical supplies - Parasitological confirmation	25,536 $	25,536 $	25,536 $	25,536 $	25,536 $	25,536 $	25,536 $	25,536 $	25,536 $	25,536 $			
Lab & medical supplies - Staging	139 $	139 $	139 $	139 $	139 $	139 $	139 $	139 $	139 $	139 $			
Lab & medical supplies - Surveillance	12,078 $	12,078 $	12,078 $	12,078 $	12,078 $	12,078 $	12,078 $	12,078 $	12,078 $	12,078 $			
Human Resources	31,074 $	31,074 $	31,074 $	31,074 $	31,074 $	31,074 $	31,074 $	31,074 $	31,074 $	31,074 $			
Other supplies and materials	5,615 $	5,615 $	5,615 $	5,615 $	5,615 $	5,615 $	5,615 $	5,615 $	5,615 $	5,615 $			
Fuel cost	5,719 $	5,719 $	5,719 $	5,719 $	5,719 $	5,719 $	5,719 $	5,719 $	5,719 $	5,719 $			
Management	37,725 $	29,317 $	30,484 $	29,359 $	30,759 $	30,429 $	30,526 $	35,128 $	30,759 $	29,359 $		**5 Year**	**10 Year**
**Total Screening**	**228,191 $**	**163,731 $**	**172,678 $**	**164,053 $**	**174,790 $**	**172,261 $**	**172,999 $**	**208,282 $**	**174,790 $**	**164,053 $**	**Min**	163,731 $	163,731 $
Cost per person screened	3.46 $	2.48 $	2.62 $	2.49 $	2.65 $	2.61 $	2.62 $	3.16 $	2.65 $	2.49 $	**Max**	228,191 $	228,191 $
Cost per person case detected	30,905 $	22,175 $	23,387 $	22,219 $	23,673 $	23,330 $	23,430 $	28,209 $	23,673 $	22,219 $	**Average**	179,789 $	179,583 $

	Year 1	Year 2	Year 3	Year 4	Year 5	Year 6	Year 7	Year 8	Year 9	Year 10			
	**Mini team**												
**Costs at the mobile team level**													
**Capital Equipment**	**35,921 $**	**0 $**	**6,605 $**	**1,480 $**	**6,605 $**	**4,035 $**	**31,885 $**	**0 $**	**6,605 $**	**1,480 $**			
Vehicle/motorcycles	23,800 $	0 $	0 $	0 $	0 $	0 $	23,800 $	0 $	0 $	0 $			
Medical and laboratory equipment	2,848 $	0 $	2,005 $	0 $	2,005 $	843 $	2,005 $	0 $	2,005 $	0 $			
Energy source	1,294 $	0 $	0 $	1,294 $	0 $	0 $	1,294 $	0 $	0 $	1,294 $			
Electronics	3,193 $	0 $	0 $	0 $	0 $	3,193 $	0 $	0 $	0 $	0 $			
Other equipment	1,086 $	0 $	900 $	186 $	900 $	0 $	1,086 $	0 $	900 $	186 $			
Training	3,700 $	0 $	3,700 $	0 $	3,700 $	0 $	3,700 $	0 $	3,700 $	0 $			
**Annual Recurrent costs**	**136,005 $**	**130,617 $**	**131,608 $**	**130,839 $**	**131,608 $**	**131,222 $**	**135,400 $**	**130,617 $**	**131,608 $**	**130,839 $**			
Lab & medical supplies - Screening tests	57,934 $	57,934 $	57,934 $	57,934 $	57,934 $	57,934 $	57,934 $	57,934 $	57,934 $	57,934 $			
Lab & medical supplies - Parasitological confirmation	19,450 $	19,450 $	19,450 $	19,450 $	19,450 $	19,450 $	19,450 $	19,450 $	19,450 $	19,450 $			
Lab & medical supplies - Staging	0 $	0 $	0 $	0 $	0 $	0 $	0 $	0 $	0 $	0 $			
Lab & medical supplies - Surveillance	0 $	0 $	0 $	0 $	0 $	0 $	0 $	0 $	0 $	0 $			
Human Resources	20,892 $	20,892 $	20,892 $	20,892 $	20,892 $	20,892 $	20,892 $	20,892 $	20,892 $	20,892 $			
Other supplies and materials	5,045 $	5,045 $	5,045 $	5,045 $	5,045 $	5,045 $	5,045 $	5,045 $	5,045 $	5,045 $			
Fuel cost	2,299 $	2,299 $	2,299 $	2,299 $	2,299 $	2,299 $	2,299 $	2,299 $	2,299 $	2,299 $			
Management	30,386 $	24,998 $	25,988 $	25,220 $	25,988 $	25,603 $	29,780 $	24,998 $	25,988 $	25,220 $		**5 Year**	**10 Year**
**Total Screening**	**171,926 $**	**130,617 $**	**138,213 $**	**132,319 $**	**138,213 $**	**135,258 $**	**167,285 $**	**130,617 $**	**138,213 $**	**132,319 $**	**Min**	130,617 $	130,617 $
Cost per person screened	2.60 $	1.98 $	2.09 $	2.00 $	2.09 $	2.05 $	2.53 $	1.98 $	2.09 $	2.00 $	**Max**	171,926 $	171,926 $
Cost per person case detected	26,120 $	19,844 $	20,998 $	20,102 $	20,998 $	20,549 $	25,414 $	19,844 $	20,998 $	20,102 $	**Average**	139,813 $	141,498 $

Table 3 Financial costs by year (discount rate 3%)

For each year (n) in the future the value of costs was multiplied by $1/(1+D)^n$ where D is the discount rate.

	Year 1	Year 2	Year 3	Year 4	Year 5	Year 6	Year 7	Year 8	Year 9	Year 10			
	**Traditional Team**												
**Costs at the mobile team level**													
**Capital Equipment**	**56,052 $**	**0 $**	**7,333 $**	**256 $**	**8,544 $**	**6,398 $**	**6,749 $**	**31,499 $**	**7,591 $**	**214 $**			
Vehicle/motorcycles	38,740 $	0 $	0 $	0 $	0 $	0 $	0 $	31,499 $	0 $	0 $			
Medical and laboratory equipment	5,181 $	0 $	2,997 $	0 $	2,825 $	1,726 $	2,663 $	0 $	2,510 $	0 $			
Energy source	1,821 $	0 $	0 $	0 $	0 $	1,571 $	0 $	0 $	0 $	0 $			
Electronics	3,595 $	0 $	0 $	0 $	0 $	3,101 $	0 $	0 $	0 $	0 $			
Other equipment	3,016 $	0 $	848 $	256 $	2,431 $	0 $	988 $	0 $	2,160 $	214 $			
Training	3,700 $	0 $	3,488 $	0 $	3,287 $	0 $	3,099 $	0 $	2,921 $	0 $			
**Annual Recurrent costs**	**172,139 $**	**158,962 $**	**155,432 $**	**149,876 $**	**146,755 $**	**142,196 $**	**138,135 $**	**137,853 $**	**130,390 $**	**125,518 $**			
Lab & medical supplies - Screening tests	54,253 $	52,673 $	51,139 $	49,649 $	48,203 $	46,799 $	45,436 $	44,113 $	42,828 $	41,581 $			
Lab & medical supplies - Parasitological confirmation	25,536 $	24,793 $	24,070 $	23,369 $	22,689 $	22,028 $	21,386 $	20,763 $	20,159 $	19,571 $			
Lab & medical supplies - Staging	139 $	135 $	131 $	128 $	124 $	120 $	117 $	113 $	110 $	107 $			
Lab & medical supplies - Surveillance	12,078 $	11,726 $	11,384 $	11,053 $	10,731 $	10,418 $	10,115 $	9,820 $	9,534 $	9,256 $			
Human Resources	31,074 $	30,169 $	29,290 $	28,437 $	27,609 $	26,805 $	26,024 $	25,266 $	24,530 $	23,816 $			
Other supplies and materials	5,615 $	5,451 $	5,293 $	5,139 $	4,989 $	4,844 $	4,702 $	4,566 $	4,433 $	4,303 $			
Fuel cost	5,719 $	5,552 $	5,391 $	5,234 $	5,081 $	4,933 $	4,790 $	4,650 $	4,515 $	4,383 $			
Management	37,725 $	28,463 $	28,734 $	26,868 $	27,329 $	26,249 $	25,565 $	28,562 $	24,282 $	22,501 $		**5 Year**	**10 Year**
**Total Screening**	**228,191 $**	**158,962 $**	**162,765 $**	**150,131 $**	**155,298 $**	**148,594 $**	**144,884 $**	**169,352 $**	**137,981 $**	**125,733 $**	**Min**	150,131 $	125,733 $
Cost per person screened	3.46 $	2.41 $	2.47 $	2.27 $	2.35 $	2.25 $	2.20 $	2.57 $	2.09 $	1.91 $	**Max**	228,191 $	228,191 $
Cost per person case detected	30,905 $	21,529 $	22,044 $	20,333 $	21,033 $	20,125 $	19,622 $	22,936 $	18,687 $	17,029 $	**Average**	171,070 $	158,189 $

	**Mini team**												
**Costs at the mobile team level**													
**Capital Equipment**	**35,921 $**	**0 $**	**6,226 $**	**1,355 $**	**5,869 $**	**3,481 $**	**26,704 $**	**0 $**	**5,214 $**	**1,135 $**			
Vehicle/motorcycles	23,800 $	0 $	0 $	0 $	0 $	0 $	19,932 $	0 $	0 $	0 $			
Medical and laboratory equipment	2,848 $	0 $	1,890 $	0 $	1,782 $	727 $	1,679 $	0 $	1,583 $	0 $			
Energy source	1,294 $	0 $	0 $	1,184 $	0 $	0 $	1,084 $	0 $	0 $	992 $			
Electronics	3,193 $	0 $	0 $	0 $	0 $	2,754 $	0 $	0 $	0 $	0 $			
Other equipment	1,086 $	0 $	848 $	171 $	800 $	0 $	910 $	0 $	710 $	143 $			
Training	3,700 $	0 $	3,488 $	0 $	3,287 $	0 $	3,099 $	0 $	2,921 $	0 $			
**Annual Recurrent costs**	**136,005 $**	**126,813 $**	**124,053 $**	**119,736 $**	**116,932 $**	**113,193 $**	**113,395 $**	**106,204 $**	**103,892 $**	**100,277 $**			
Lab & medical supplies - Screening tests	57,934 $	56,246 $	54,608 $	53,018 $	51,473 $	49,974 $	48,519 $	47,105 $	45,733 $	44,401 $			
Lab & medical supplies - Parasitological confirmation	19,450 $	18,884 $	18,334 $	17,800 $	17,281 $	16,778 $	16,289 $	15,815 $	15,354 $	14,907 $			
Lab & medical supplies - Staging	0 $	0 $	0 $	0 $	0 $	0 $	0 $	0 $	0 $	0 $			
Lab & medical supplies - Surveillance	0 $	0 $	0 $	0 $	0 $	0 $	0 $	0 $	0 $	0 $			
Human Resources	20,892 $	20,283 $	19,692 $	19,119 $	18,562 $	18,021 $	17,497 $	16,987 $	16,492 $	16,012 $			
Other supplies and materials	5,045 $	4,898 $	4,755 $	4,617 $	4,482 $	4,352 $	4,225 $	4,102 $	3,983 $	3,867 $			
Fuel cost	2,299 $	2,232 $	2,167 $	2,103 $	2,042 $	1,983 $	1,925 $	1,869 $	1,814 $	1,762 $			
Management	30,386 $	24,270 $	24,497 $	23,080 $	23,090 $	22,085 $	24,941 $	20,325 $	20,516 $	19,329 $		**5 Year**	**10 Year**
**Total Screening**	**171,926 $**	**126,813 $**	**130,279 $**	**121,091 $**	**122,800 $**	**116,674 $**	**140,099 $**	**106,204 $**	**109,107 $**	**101,412 $**	**Min**	121,091 $	101,412 $
Cost per person screened	2.60 $	1.92 $	1.97 $	1.83 $	1.86 $	1.77 $	2.12 $	1.61 $	1.65 $	1.54 $	**Max**	171,926 $	171,926 $
Cost per person case detected	26,120 $	19,266 $	19,792 $	18,397 $	18,656 $	17,726 $	21,284 $	16,135 $	16,576 $	15,407 $	**Average**	134,582 $	124,640 $
