# Supplementary material for: Cost of a new method of active screening for human African trypanosomiasis in the Democratic Republic of the Congo
Source: PLoS Negl Trop Dis. 2020 Dec 14;14(12):e0008832. doi: 10.1371/journal.pntd.0008832 (PMC7769601; doi:10.1371/journal.pntd.0008832)
Supplement: S2 Table — (PDF) [file pntd.0008832.s002.pdf]

S2 Economic costs

The annual cost per item was based the invoices for replacement equipment in 2017 – 2018, observations, training budgets and past training expenses, discussions with PNLTHA experts, PNLTHA procurement registers and WHO Choice guidelines for the useful lives of capital items.

Table 1 Details capital equipement

Category	Description	Strategy	Cost Excl VAT	Average useful life	Annual cost - Discounted			Traditional team				Mini team			
					0%	3%	5%	#	0%	3%	5%	#	0%	3%	5%
Vehicles	Toyota Land Cruiser 78 - 13 places	RDT&CATT	38,740 $	7	5,534 $	5,073 $	4,803 $	1	5,534 $	5,073 $	4,803 $	-	0 $	0 $	0 $
Vehicles	4 Motorcycles - Yamaha AG 200	RDT&CATT	5,950 $	6	1,050 $	1,034 $	988 $	-	0 $	0 $	0 $	4	4,200 $	4,136 $	3,950 $
Medical and laboratory eq.	Rotator	CATT	609 $	2	304 $	300 $	297 $	2	609 $	600 $	594 $	3	913 $	900 $	891 $
Medical and laboratory eq.	Microscope incl. accessories - 12V	RDT&CATT	843 $	5	169 $	159 $	153 $		0 $	0 $	0 $	1	169 $	159 $	153 $
Medical and laboratory eq.	Microscope incl. accessories - 220V	RDT&CATT	1,001 $	5	200 $	189 $	182 $	2	400 $	378 $	364 $	-	0 $	0 $	0 $
Medical and laboratory eq.	Haematocrit centrifuge	RDT&CATT	877 $	2	438 $	432 $	428 $	1	438 $	432 $	428 $	-	0 $	0 $	0 $
Medical and laboratory eq.	Centrifuge - 220V	RDT&CATT	337 $	2	169 $	166 $	165 $	1	169 $	166 $	165 $	-	0 $	0 $	0 $
Medical and laboratory eq.	Centrifuge - 12V	RDT&CATT	179 $	2	90 $	88 $	88 $	-	0 $	0 $	0 $	1	90 $	88 $	88 $
Medical and laboratory eq.	Micropipette	RDT&CATT	374 $	2	187 $	184 $	183 $	2	374 $	369 $	365 $	-	0 $	0 $	0 $
Electronics	Laptop	RDT&CATT	1,200 $	5.4	222 $	208 $	200 $	1	222 $	208 $	200 $	-	0 $	0 $	0 $
Electronics	PDA & camera incl. accessories	RDT&CATT	798 $	5.4	148 $	139 $	133 $	3	443 $	416 $	399 $	4	591 $	554 $	532 $
Energy source	Generator incl. accessories	RDT&CATT	1,821 $	5	364 $	344 $	331 $	1	364 $	344 $	331 $		0 $	0 $	0 $
Energy source	Solar panel incl. accessories	RDT&CATT	323 $	3	108 $	105 $	103 $	-	0 $	0 $	0 $	4	431 $	419 $	411 $
Other equipment	Tents	RDT&CATT	1,836 $	4	459 $	439 $	427 $	1	459 $	439 $	427 $	-	0 $	0 $	0 $
Other equipment	Camping beds	RDT&CATT	47 $	3	16 $	15 $	15 $	6	93 $	90 $	89 $	4	62 $	60 $	59 $
Other equipment	Miscellaneous	RDT&CATT	900 $	2	450 $	443 $	439 $	1	450 $	443 $	439 $	1	450 $	443 $	439 $
Training	Training	RDT&CATT	3,700 $	2	1,850 $	1,823 $	1,806 $	1	1,850 $	1,823 $	1,806 $	1	1,850 $	1,823 $	1,806 $

Table 2 Overview capital equipement

Summary		Traditional team			Mini team		
Type	Category	0%	3%	5%	0%	3%	5%
Capital Equipment	Vehicles	5,543 $	5,073 $	4,803 $	4,200 $	4,136 $	3,950 $
Capital Equipment	Medical and laboratory eq.	1,990 $	1,944 $	1,916 $	1,171 $	1,147 $	1,132 $
Capital Equipment	Energy source	364 $	344 $	331 $	431 $	419 $	411 $
Capital Equipment	Electronics	666 $	624 $	599 $	591 $	554 $	532 $
Capital Equipment	Other equipment	1,002 $	973 $	955 $	512 $	504 $	498 $
Capital Equipment	Training	1,850 $	1,823 $	1,806 $	1,850 $	1,823 $	1,806 $
	**Total Equipment team using CATT**	**11,406 $**	**10,782 $**	**10,411 $**	**8,756 $**	**8,582 $**	**8,330 $**
Capital Equipment	Medical and laboratory eq. Excluding CATT rotators	- 609 $	- 600 $	- 594 $	-913 $	- 900 $	- 891 $
	**Total Equipment team using RDT**	**10,798 $**	**10,182 $**	**9,817 $**	**7,843 $**	**7,683 $**	**7,438**

The annualised discounted economic cost of the equipment was calculated by averaging the annual discounted costs based on the useful life of the equipment. For each year (n) in the future the value of costs was multiplied by $1/(1+D)^n$ where D is the discount rate.

For example for a car with a value of 40,000$ with a useful life of 4 years and a discount rate of 3% the annual discounted economic cost would be calculated as followed:

$$[10,000 * (1/(1+0.03)^0+1/(1+0.03)^1+1/(1+0.03)^2+/(1+0.03)^3)]/4$$

$$[10,000 * (1+0.97+0.94+0.92)]/4= 38,286 /4 = 9,571$$

Table 3 Details estimation of the cost of consumables per test

The cost per test was based on the observations regarding the consumables used during screening activities and market prices during the project.

Description	Packaging	# Units/packaging	Price	Currency	Unit price Excl VAT	Imported	Unit price @ Kinshasa	Non-specific Annual consumption	CATT	Lymph node aspiration	Blood sample	CTC	meat	Lumbar puncture examination	CATT titration	RDT SD	RDT Sero -K-
									The average number of items used per test								
Cotton balls	Roll	1	7.00	$	$ 7.00		$ 7.00	36									
Providone - Disinfectant	250 ml	1	5.00	$	$ 5.00		$ 5.00	36									
Gloves	Box	100	7.00	$	$ 0.07		$ 0.07	3,000									
Bin	Piece	1	20.00	$	$ 20.00		$ 20.00	36									
Kit CATT	Kit CATT	1	0.52	Euro	$ 0.61	x	$ 0.67		1						5		
Lancet	Box	200	3.10	Euro	$ 0.02	x	$ 0.02		1								
Heparinized capillary tubes	Box	100	3.03	Euro	$ 0.04	x	$ 0.04		1			1					
Bulb for capillary tubes	Box	100	1.42	Euro	$ 0.02	x	$ 0.02		0.5								
hypodermic needle	Box	100	8.00	$	$ 0.08		$ 0.08			1							
Syringe 5cc	Box	100	7.00	$	$ 0.07		$ 0.07			1				1			
Tropicalized microscope slide	Box	50	4.00	$	$ 0.08		$ 0.08			1		1					
Cover glass	Box	100	2.00	$	$ 0.02		$ 0.02			1							
Gauze	Box	10	3.50	$	$ 0.35		$ 0.35				0.5						
Adapter vacutainer Tubes	Box	1	0.50	$	$ 0.50		$ 0.50				0.5						
Vacutainer needle	Box	1	0.50	$	$ 0.50		$ 0.50				1						
Heparinized vacutainer tubes	Box	100	35.00	$	$ 0.35		$ 0.35				1						
Plasticine	Sheet	6	30.00	$	$ 5.00		$ 5.00					0.03					
Specialized cover glass	Box	10	15.00	$	$ 1.50		$ 1.50					1		1			
Kit mAECT	Box	1	3.50	Euro	$ 4.13		$ 4.13						1				
Lumbar puncture needle	Piece	1	1.30	$	$ 1.30		$ 1.30							1			
Modified single centrifugation kit	Kit	1	10.00	Euro	$ 11.80		$ 11.80							1			
Collector tube mAECT	Box	1	3.50	Euro	$ 4.13		$ 4.13							1			
Pipette	Box	500	45.00	$	$ 0.09		$ 0.09							1	1		
Tips	Box	500	4.24	Euro	$ 0.01	x	$ 0.01								1		
Microtitration tray	Box	50	20.01	Euro	$ 0.47	x	$ 0.52								0.08		
RDT SD Bioline HAT	Box	25	13.70	$	$ 0.55	x	$ 0.60									1	
RDT HAT Sero-K-Set	Box	40	60.8	Euro	$ 1,79	X	$ 1.97										

Transport Europe - RDC: + 10% Based on estimation - ITM exports 2017

Exchange rate euro - $ 1.180 Average exchange rate of 2018

Table 4 Total cost of supplies and material per test

Test	Price subsidized
Non-specific => All CATT or RDT	$ 0.02
CATT	$ 0.74
Lymph node aspiration (LNA)	$ 0.25
Blood sample (BS) => To be taken Once for CTC or mAECT	$ 1.28
CTC	$ 1.77
mAECT	$ 4.13
Lumbar puncture examination (LP)	$ 18.89
CATT titration	$ 3.52
RDT SD Bioline HAT	$ 0.60
RDT HAT Sero-K-Set	$ 1,97

Table 5 Total number of tests performed and HAT cases detected per team

Type of team	CATT	CATT +	LNA	CTC	mEACT	HAT stage 1	HAT stage 2
Traditional Mobile Team	65,190	276 (0,4%)	17	220	271	4	7
Average Mini Mobile Teams	66,480	833 (1%)	137	-	795	2	4
Mini Mobile Team 1	67,715	839	242	-	811	1	1
Mini Mobile Team 2	61,958	806	60	-	772	5	10
Mini Mobile Team 3	69,767	855	108	-	801		
**Average all Mobile Teams**	**65,835**	555	**77**	**110**	**533**	**3**	**5**

Table 6 Percentage of tests discarded per team

Test	Traditional Team	Mini Team
% of CATT discarded	7.5%	15%

Table 7 Annual recurrent costs for HAT screening & Parasitological confirmation

				Traditional Team		Mini Team	
**Category**	**Description**	**Screening**	**Cost excl VAT**	**#**	**Cost**	**#**	**Cost**
**Annual Recurrent costs**							
Screening	CATT & non-specific costs	CATT	0.77 $	65,190	49,952 $	66,480	50,940 $
Screening	CATT discarded	CATT	0.74 $	4,889	3,635 $	9,972	7,415 $
Parasitological confirmation	LNA	CATT	0.25 $	17	4 $	137	34 $
Parasitological confirmation	Blood sample	CATT	1.28 $	220	280 $	795	1,013 $
Parasitological confirmation	CTC	CATT	1.77 $	220	389 $		0 $
Parasitological confirmation	mAECT	CATT	4.13 $	271	1,119 $	795	3,282 $
Staging	LP	CATT	18.89 $	11	208 $		0 $
Surveillance	CATT titration	CATT	3.52 $	265	932 $	-	0 $
					56,521$		62,6284 $

Exchange rate euro - $ 1.180 Average exchange rate of 2018

Table 8 Total annual recurrent costs for HAT screening & Parasitological confirmation per test

Summary		Traditional team	Mini team
**Type**	**Category**	**Cost**	**Cost**
Annual Recurrent costs	Screening	53,587 $	58,355 $
Annual Recurrent costs	Parasitological confirmation	1.793 $	4,329 $
Annual Recurrent costs	Staging	208 $	0 $
Annual Recurrent costs	Surveillance	932 $	0 $
		56,521 $	62,684 $

Table 9 Total annual recurrent costs related to Human Resources (HR)

The annual cost per item was based on the invoices, monthly wage statements, discussions with PNLTHA experts and PNLTHA procurement registers.

Category	Description	Screening	Traditional Team	Mini Team
HR	Salaries & subsidies	RDT&CATT	2,886 $	1,066 $
HR	Fixed premium	RDT&CATT	11,460 $	11,816 $
HR	Variable premium	RDT&CATT	16,728 $	4,536 $
HR	Allowance local health care workers	RDT&CATT	0 $	3,475 $

Exchange rate euro - $ 1.180 Average exchange rate of 2018

Summary		Traditional team	Mini team
Type	Category	Average	Average
Annual Recurrent costs	HR	31,074 $	20,892 $

Table 10 Total Annual recurrent costs – Other supplies and materials

The annual cost per item was based on the invoices, monthly wage statements, discussions with PNLTHA experts and PNLTHA procurement registers.

				Traditional Team		Mini Team	
Category	Description	Screening	Cost excl VAT	#	Average	#	Average
**Annual Recurrent costs**							
Operation & maintenance vehicles	Tyres, batterie, insurance, etc.	RDT&CATT	1,225 $	1	1,225 $	1	1,225 $
Operation & maintenance vehicles	Maintenance	RDT&CATT	2,000 $	1	2,000 $	1	1,000 $
Medical and laboratory eq.	Other lab supplies and material	RDT&CATT	500 $	1	500 $	1	500 $
Energy source	Batteries incl. accessories	RDT&CATT	250 $	1	250 $	4	1,000 $
Other equipment and consumables	Other Camping Equipment	RDT&CATT	80 $	8	640 $	4	320 $
Other equipment and consumables	Other operational costs	RDT&CATT	1,000 $	1	1,000 $	1	1,000 $

Exchange rate euro - $ 1.180 Average exchange rate of 2018

Summary		Traditional team	Mini team
Type	Category	Average	Average
Annual Recurrent costs	Operation & maintenance vehicles	3,225 $	2,225 $
Annual Recurrent costs	Medical and laboratory eq.	500 $	500 $
Annual Recurrent costs	Energy source	250 $	1,000 $
Annual Recurrent costs	Other equipment and consumables	1,640 $	1,320 $
Annual Recurrent costs	Other Supplies and materials	5,615 $	5,045 $

Table 11 Total Annual recurrent costs – Fuel

The average fuel cost was estimated through trimestral reports of mobile teams, car and motorcycle logbooks, fuel invoices. The fuel price range for the DRC was based on invoices and discussions with PNLTHA.

	Traditional team		Mini team
Consumption - l/ 100 km	27	Consumption - l/ 100 km	8
Number of KM - Screening	5,179	Number of KM - Screening	17,306
Number of KM - Coordination	5,482	Number of KM - Coordination	-
Number of KM - Monitoring	1,644	Number of KM - Monitoring	651
Annual consumption vehicle (l)	3,322	Annual consumption mini team (l)	1,437
Annual consumption generator	252	Annual consumption generator	
Price diesel - l	1.6 $	Price diesel - l	1.6 $
**Total fuel cost**	**5,719 $**	**Total fuel cost**	**2,299 $**

Summary		Traditional team	Mini team
**Type**	**Category**	**Average**	**Average**
Annual Recurrent costs	Fuel cost	5,719 $	2,299 $

Table 12 Total Annual recurrent costs – Management costs

The estimation of PNLTHA's direct management costs was based on the PNLTHA budgets and discussions with the PNLTHA.

Provincial Level - PNLTHA	Unit price	Units	Annual cost	Cost per mobile team	Source of information
**Management provincial coordination PNLTHA**				**3,398 $**	5% Annual cost
Coordinations - Investments cars	39,500 $	0	5,411 $	271 $	
Provincial coordinator	1,000 $	1	12,000 $	600 $	
Supervisor	600 $	1	7,200 $	360 $	
Administrator	450 $	1	5,400 $	270 $	
Data manager	300 $	1	3,600 $	180 $	
Logistics	450 $	1	5,400 $	270 $	
Chauffeur	300 $	1	3,600 $	180 $	
Surveillant	150 $	4	7,200 $	360 $	
Christmas bonus	100 $	10	1,000 $	50 $	Estimation annual costs based on interviews and budget PNLTHA
Working cost - office supplies - 1 650 $/trimester	1,650 $	4	6,600 $	330 $	The provincial coordination spends around 70% of their time on the management of active screening
Communication - 100$/month	100 $	12	1,200 $	60 $	activities in 15 health zones
Internet - 55 $	300 $	12	3,600 $	180 $	
Maintenance car, new tires, etc.- 750$/Trim	750 $	4	3,000 $	150 $	
Installation cost	11,000 $	25%	2,750 $	138 $	
**Annual planning meeting at Provincial level (ECP, ECZS, UM)**				**674 $**	
Transport	2,750 $	1	2,750 $	138 $	
Meeting room, Hotel, lunch	6,600 $	1	6,600 $	330 $	
Per Diem	3,575 $	1	3,575 $	179 $	
Miscellaneous	550 $	1	550 $	28 $	
**Provincial Level: 1 supervision/trimester - 2 people - 5 days**				**2,235 $**	Total cost 4 visits annually
Fuel -21l/100km - 470km + 300km return Kikwit- Health zone	2 $	647	1,035 $	1,035 $	Estimation annual costs based on interviews and budget PNLTHA
Per diem - 2 people - 6 days	10 $	40	400 $	400 $	1 visit per trimester per health zone
Lodging - 2 people - 5 nights	20 $	40	800 $	800 $	
**Total Provincial Level PNLTHA: Management & Supervision**				**6,307 $**	

Management central level PNLTHA	Unit price	Units	Annual cost	Cost per mobile team	Source of information
**Management**				**795 $**	**2.5% of annual cost**
Coordinator	2,000 $	1	12,000 $	300 $	
Data management	550 $	1	6,600 $	165 $	Estimation annual costs based on interviews and budget PNLTHA
Lab supervisor	1,000 $	1	6,000 $	150 $	Project specific management team at central level that divides their time over 2 coordinations
Financial manager	1,000 $	0	3,600 $	90 $	
Logistics assistant	1,000 $	0	3,600 $	90 $	
**National Level: 1 supervision every trimester - 3 people - 5 days**				**859 $**	**5% of annual cost (see above: 70% of the activities active screening spread over 15 health zones)**
Travel costs	2,000 $	4	8,000 $	400 $	Estimation annual costs based on interviews and budget PNLTHA; 1 visit per trimester per
Per diem - 2 people - 9 days	85 $	108	9,180 $	459 $	coordination
**Total national Level PNLTHA: Management & Supervision**				**1,654 $**	
**Total Management cost**				**7,961 $**	

Summary		Traditional team	Mini team
**Type**	**Category**	**Average**	**Average**
Annual Recurrent costs	Management	7,691 $	7,691 $
Annual Recurrent costs	Management	+ 15% overall costs	+15% overall costs

Details calculation

Total annual cost excluding variable management cost =

Cost capital equipment

\+ # People screened x non-specific test costs

\+ # People screened x (1+% discarded tests) x cost serological test

\+ # positive tests x cost confirmation tests (LNA + BS + CTC + mAECT for traditional team or LNA + BS + mAECT for a mini team)

\+ # Cases x LP

\+ (N - # Cases) x cost CATT titration (only traditional teams)

\+ Cost Human Resources

\+ Cost Other supplies and materials

\+ Cost Fuel

\+ Cost Fixed management

Total annual cost =

Total annual cost excluding variable management cost x 1.15
