# Supplementary material for: Cost of a new method of active screening for human African trypanosomiasis in the Democratic Republic of the Congo
Source: PLoS Negl Trop Dis. 2020 Dec 14;14(12):e0008832. doi: 10.1371/journal.pntd.0008832 (PMC7769601; doi:10.1371/journal.pntd.0008832)
Supplement: S3 Table — (PDF) [file pntd.0008832.s003.pdf]

S3 Scenario analysis: Baseline scenario with both teams operational in a similar setting

Table 1 Baseline scenario

Assumptions	Baseline
Population screened per year per team (N)	66,000
Prevalence	0.011%
Number of cases	9
Sensitivity CATT	0.953
Number of confirmation (overall results 2,776 / 264,6930) (Confirmation)	1.05% CATT tests positive
Number of confirmations	3,440
Sensitivity algorithm LNA-CTC-mAECT (Traditional team)	0.903
Sensitivity algorithm LNA-mAECT (Mini team)	0.805
Number of cases identified: LNA-mAECT (Mini Team)	6.6
Number of cases identified: LNA CTC mAECT (Traditional team)	7.4
% of CATT discarded Traditional Team	8%
% of CATT discarded Mini Team	15%
% of RDT discarded Traditional & Mini Team	5%
Non-specific => All CATT or RDT	0.02 $
CATT	0.74 $
Lymph node aspiration (LNA)	0.25 $
Blood sample (BS)	1.28 $
CTC	1.77 $
mAECT	4.13 $
Lumbar punction examination (LP)	18.89 $
CATT titration	3.52 $

	Traditional Team		Mini team		Impact price per person screened	
**Capital Equipment**	**10,782**	**7.6%**	**8,582**	**7.0%**	**0.03**	**11.6%**
**Annual Recurrent costs**	**130,751**	**92.4%**	**114,039**	**93.0%**	**0.25**	**88.4%**
Lab & medical supplies - Screening tests (CATT & Discarded CATT)	61,922	44.8%	61,849	50.4%	0.00	0.4%
Lab & medical supplies - Parasitological confirmation	54,253	38.3%	57,934	47.2%	-0.06	-19.5%
Lab & medical supplies - Staging	5,140	3.6%	3,915	3.2%	0.02	6.5%
Lab & medical supplies - Surveillance	114	0.1%	-	0.0%	0.00	0.6%
Human Resources	2,415	1.7%	-	0.0%	0.04	12.8%
Other supplies and materials	5,615	4.0%	5,045	4.1%	0.01	3.0%
Fuel cost	5,719	4.0%	2,299	1.9%	0.05	18.1%
Management	26,421	18.7%	23,955	19.5%	0.04	13.0%
**Total Screening**	**141,533**	**100%**	**122,621**	**100%**		
Cost per person screened	2.14		1.86		0.29	15.4%
Cost per person case detected	23,551		22,888		663	2.9%
