# Supplementary material for: Cost of a new method of active screening for human African trypanosomiasis in the Democratic Republic of the Congo
Source: PLoS Negl Trop Dis. 2020 Dec 14;14(12):e0008832. doi: 10.1371/journal.pntd.0008832 (PMC7769601; doi:10.1371/journal.pntd.0008832)
Supplement: S4 Table — (PDF) [file pntd.0008832.s004.pdf]

1. Diagnostic test and epidemiological parameters used for scenario analysis and sensitivity analysis

Table 1 Diagnostic test and epidemiological parameters used for model and sensitivity analysis

**Epidemiological parameters**	Baseline	Sensitivity analysis		Reference
Population screened per year per team (n)	66,000	54,000	72,000	[1]
HAT prevalence (%)	0.011%	0.005%	0.030%	[1]
**Diagnostic parameters**				
Specificity CATT		0.835	0.993	[2,3]
Sensitivity CATT	0.953			[2,3]
Specificity RDT SD Bioline HAT	0.879			[4]
Sensitivity RDT SD Bioline HAT	0.996			[4]
Specificity RDT HAT Sero-K-Set	0.883			[4]
Sensitivity RDT HAT Sero-K-Set	0.991			[4]
Sensitivity algorithm LNA-CTC-mAECT	0.903			[5]
Sensitivity algorithm LNA-mAECT	0.802			[5]
**Cost parameters**				
Vehicle/Motorcycle use life	7y/5.6y	3y/2y	10y/10y	[6]
Cost mAECT (US$)	4,13	0	8.26	
Fuel Cost (75% - 150%)	100%	75%	150%	
Discount rate	3%	0%	5%	[7]
**Characteristics of mobile teams**				
% of HAT suspects who are microscopically tested	100			[1]

References

1. Programme national de lutte contre la trypanosomiase humaine africaine en DRC (PNLTHA). TrypElim Yasa Bonga & Mosango project results [Trimestral Reports]. Kinshasa: PNLTHA; 2017 - 2018.
2. Noireau F, Gouteux JP, Duteurtre JP. Valeur diagnostique du test d'agglutination sur carte (Testryp CATT) dans le depistage de masse de la trypanosomiase humaine au Congo. Bulletin de la société de pathologie exotique. 1987;80(5):797-803, http://horizon.documentation.ird.fr/exl-doc/pleins_textes/pleins_textes_5/b_fdi_18-19/25548.pdf
3. Mitashi P, Hasker E, Lejon V, Kande V, Muyembe J-J, Lutumba P, et al. Human african trypanosomiasis diagnosis in first-line health services of endemic countries, a systematic review. PLoS Neglected Tropical Diseases. 2012;6(11):e1919-e.doi:10.1371/journal.pntd.0001919
4. Jamonneau V, Camara O, Ilboudo H, Peylhard M, Koffi M, Sakande H, et al. Accuracy of individual rapid tests for serodiagnosis of gambiense sleeping sickness in West Africa. PLoS Neglected Tropical Diseases. 2015;9(2):e0003480-e.doi:10.1371/journal.pntd.0003480
5. Lutumba P, Robays J, Miaka C, Kande V, Mumba D, Buscher P, et al. Validity, cost and feasibility of the mAECT and CTC confirmation tests after diagnosis of African of sleeping sickness. Tropical Medicine & International Health. 2006;11(4):470-8.doi:10.1111/j.1365-3156.2006.01591.x
6. World Health Organization (WHO). CHOosing Interventions that are Cost Effective (WHO-CHOICE): Table 4: Capital Item - Useful Lives Reported by Country Experts [Internet].Geneva: WHO. Updated [Cited Available from: https://www.who.int/choice/costs/prices_t4/en/
7. Severens, J.L.,Milne, R.J., Discounting Health Outcomes in Economic Evaluation: The Ongoing Debate. Value in Health.2004 ; 7(4): 397-401. doi:10.1111/j.1524-4733.2004.74002.x.

Table 2 Sensitivity analysis: Diagnostic algorithm excluding CATT titration & CTC

Assumptions	Baseline	Parameter variation	
Sensitivity algorithm LNA-CTC-mAECT (Traditional team)	0.903	0.805	
No cost included for CTC (all parasitological confirmations)	1.77 $		
No cost included for CATT titration (all HAT suspects that tested negative on HAT microscopy tests)	3.52 $		

	Diagnostic algorithm excluding CATT dilution & CTC			
	Traditional Team		Mini Team	
**Capital Equipment**	10,782 $	7.9%	8,582 $	7.0%
**Annual Recurrent costs**	126,565 $	92.1%	114,039 $	93.0%
Lab & medical supplies - Screening tests (CATT & Discarded CATT)	54,253 $	39.5%	57,934 $	47.2%
Lab & medical supplies - Parasitological confirmation	3,915 $	2.9%	3,915 $	3.2%
Lab & medical supplies - Staging	114 $	0.1%	0 $	0.0%
Lab & medical supplies - Surveillance	0 $	0.0%	0 $	0.0%
Human Resources	31,074 $	22.6%	20,892 $	17.0%
Other supplies and materials	5,615 $	4.1%	5,045 $	4.1%
Fuel cost	5,719 $	4.2%	2,299 $	1.9%
Management	25,875 $	18.8%	23,955 $	19.5%
**Total Screening**	137,347 $	100%	122,621 $	100%
Cost per person screened	2.08 $		1.86 $	
Cost per person case detected	25,637 $		22,888 $	

Table 3 Sensitivity analysis: Discount rate 5% - 0%

Assumptions	Baseline	Parameter variation	
	3%	5%	0%

	Discount rate: 5%				Discount rate: 0%			
	Traditional Team		Mini Team		Traditional Team		Mini Team	
**Capital Equipment**	10,411 $	7.4%	8,330 $	6.8%	**11,406 $**	**8.0%**	**8,756 $**	**7.1%**
**Annual Recurrent costs**	130,720 $	92.6%	114,001 $	93.2%	**130,869 $**	**92.0%**	**114,065 $**	**92.9%**
Lab & medical supplies - Screening tests (CATT & Discarded CATT)	54,253 $	38.4%	57,934 $	47.4%	54,253 $	38.1%	57,934 $	47.2%
Lab & medical supplies - Parasitological confirmation	5,140 $	3.6%	3,915 $	3.2%	5,140 $	3.6%	3,915 $	3.2%
Lab & medical supplies - Staging	139 $	0.1%	0 $	0.0%	139 $	0.1%	0 $	0.0%
Lab & medical supplies - Surveillance	2,410 $	1.7%	0 $	0.0%	2,410 $	1.7%	0 $	0.0%
Human Resources	31,074 $	22.0%	20,892 $	17.1%	31,074 $	21.8%	20,892 $	17.0%
Other supplies and materials	5,615 $	4.0%	5,045 $	4.1%	5,615 $	3.9%	5,045 $	4.1%
Fuel cost	5,719 $	4.1%	2,299 $	1.9%	5,719 $	4.0%	2,299 $	1.9%
Management	26,369 $	18.7%	23,917 $	19.6%	26,518 $	18.6%	23,981 $	19.5%
**Total Screening**	141,130 $	100%	122,330 $	100%	**142,275 $**	**100%**	**122,820 $**	**100%**
Cost per person screened	2.14 $		1.85 $		2.16 $		1.86 $	
Cost per person case detected	19,114 $		18,585 $		19,269 $		18,659 $	

Table 4 Sensitivity analysis: HAT Prevalence 0.005 % - 0.030%

Assumptions	Baseline	Parameter variation	
Prevalence	0.011%	0.005%	0.030%

	Prevalence – 0.005%				Prevalence - 0.030%			
	Traditional Team		Mini Team		Traditional Team		Mini Team	
**Capital Equipment**	**10,782 $**	**7.6%**	**8,582 $**	**7.0%**	**10,782 $**	**7.3%**	**8,582 $**	**6.6%**
**Annual Recurrent costs**	**130,695 $**	**92.4%**	**114,039 $**	**93.0%**	**137,712 $**	**92.7%**	**120,805 $**	**93.4%**
Lab & medical supplies - Screening tests (CATT & Discarded CATT)	54,253 $	38.3%	57,934 $	47.2%	54,253 $	36.5%	57,934 $	44.8%
Lab & medical supplies - Parasitological confirmation	5,140 $	3.6%	3,915 $	3.2%	5,140 $	3.5%	3,915 $	3.0%
Lab & medical supplies - Staging	54 $	0.0%	0 $	0.0%	322 $	0.2%	0 $	0.0%
Lab & medical supplies - Surveillance	2,426 $	1.7%	0 $	0.0%	2,376 $	1.6%	0 $	0.0%
Human Resources	31,074 $	22.0%	20,892 $	17.0%	31,074 $	20.9%	20,892 $	16.1%
Other supplies and materials	5,615 $	4.0%	5,045 $	4.1%	5,615 $	3.8%	5,045 $	3.9%
Fuel cost	5,719 $	4.0%	2,299 $	1.9%	5,719 $	3.9%	2,299 $	1.8%
Management	26,414 $	18.7%	23,955 $	19.5%	33,213 $	22.4%	30,721 $	23.7%
**Total Screening**	**141,477 $**	**100%**	**122,621 $**	**100%**	**148,494 $**	**100%**	**129,388 $**	**100%**
Cost per person screened	2.14 $		1.86 $		2.25 $		1.96 $	
Cost per person case detected	49,818 $		48,435 $		8,715 $		8,518 $	

Table 5 Sensitivity analysis: Fuel cost 75% - 150% initial cost

Assumptions	Baseline	Parameter variation	
Fuel cost Traditional team	5,719 $	- 25%	+50%
Fuel cost Mini Team	2,299 $	- 25%	+50%

	Fuel cost – 25%				Fuel cost + 50%			
	Traditional Team		Mini Team		Traditional Team		Mini Team	
**Capital Equipment**	**10,782 $**	**7.7%**	**8,582 $**	**7.0%**	**10,782 $**	**7.4%**	**8,582 $**	**6.9%**
**Annual Recurrent costs**	**129,107 $**	**92.3%**	**113,378 $**	**93.0%**	**134,039 $**	**92.6%**	**115,360 $**	**93.1%**
Lab & medical supplies - Screening tests (CATT & Discarded CATT)	54,253 $	38.8%	57,934 $	47.5%	54,253 $	37.5%	57,934 $	46.7%
Lab & medical supplies - Parasitological confirmation	5,140 $	3.7%	3,915 $	3.2%	5,140 $	3.5%	3,915 $	3.2%
Lab & medical supplies - Staging	114 $	0.1%	0 $	0.0%	114 $	0.1%	0 $	0.0%
Lab & medical supplies - Surveillance	2,415 $	1.7%	0 $	0.0%	2,415 $	1.7%	0 $	0.0%
Human Resources	31,074 $	22.2%	20,892 $	17.1%	31,074 $	21.5%	20,892 $	16.9%
Other supplies and materials	5,615 $	4.0%	5,045 $	4.1%	5,615 $	3.9%	5,045 $	4.1%
Fuel cost	4,289 $	3.1%	1,724 $	1.4%	8,578 $	5.9%	3,448 $	2.8%
Management	26,207 $	18.7%	23,869 $	19.6%	26,850 $	18.5%	24,127 $	19.5%
**Total Screening**	**139,888 $**	**100%**	**121,960 $**	**100%**	**144,821 $**	**100%**	**123,943 $**	**100%**
Cost per person screened	2.12 $		1.85 $		2.19 $		1.88 $	
Cost per person case detected	23,278 $		22,765 $		24,098 $		23,135 $	

Table 6 Sensitivity analysis: Vehicle/Motorcycle use life

Assumptions	Baseline	Parameter variation	
Use life vehicle	7	10	3
Use life motorcycle	5.6	10	2

	Vehicle/Motorcycle use life – 10/10				Vehicle/Motorcycle use life – 3/2			
	Traditional Team		Mini Team		Traditional Team		Mini Team	
**Capital Equipment**	**9,121 $**	**6.5%**	**6,762 $**	**5.6%**	**18,161 $**	**12.1%**	**16,282 $**	**10.9%**
**Annual Recurrent costs**	**130,502 $**	**93.5%**	**113,766 $**	**94.4%**	**131,858 $**	**87.9%**	**115,194 $**	**89.1%**
Lab & medical supplies - Screening tests (CATT & Discarded CATT)	54,253 $	38.9%	57,934 $	48.1%	54,253 $	36.2%	57,934 $	38.8%
Lab & medical supplies - Parasitological confirmation	5,140 $	3.7%	3,915 $	3.2%	5,140 $	3.4%	3,915 $	13.0%
Lab & medical supplies - Staging	114 $	0.1%	0 $	0.0%	114 $	0.1%	0 $	0.0%
Lab & medical supplies - Surveillance	2,415 $	1.7%	0 $	0.0%	2,415 $	1.6%	0 $	0.0%
Human Resources	31,074 $	22.3%	20,892 $	17.3%	31,074 $	20.7%	20,892 $	14.0%
Other supplies and materials	5,615 $	4.0%	5,045 $	4.2%	5,615 $	3.7%	5,045 $	3.4%
Fuel cost	5,719 $	4.1%	2,299 $	1.9%	5,719 $	3.8%	2,299 $	1.5%
Management	26,172 $	18.7%	23,682 $	19.6%	27,528 $	18.3%	25,110 $	18.4%
**Total Screening**	**139,623 $**	**100%**	**120,528 $**	**100%**	**150,019 $**	**100%**	**131,476 $**	**100%**
Cost per person screened	2.12 $		1.83 $		2.27 $		1.99 $	
Cost per person case detected	23,233 $		22,498 $		24,963 $		24,541 $	

Table 7 Sensitivity analysis: Cost mAECT donated 0$ - 8.26$

Assumptions	Baseline	Parameter variation	
mAECT	4.13 $	0 $	8.26 $

	mAECT donated – 0 $				mAECT gel no longer donated – 8.26 $			
	Traditional Team		Mini Team		Traditional Team		Mini Team	
**Capital Equipment**	**10,782 $**	**7.8%**	**8,582 $**	**7.2%**	**10,782 $**	**7.4%**	**8,582 $**	**6.8%**
**Annual Recurrent costs**	**127,463 $**	**92.2%**	**110,751 $**	**92.8%**	**134,039 $**	**92.6%**	**117,327 $**	**93.2%**
Lab & medical supplies - Screening tests (CATT & Discarded CATT)	54,253 $	39.2%	57,934 $	48.5%	54,253 $	37.5%	57,934 $	46.0%
Lab & medical supplies - Parasitological confirmation	2,281 $	1.6%	1,056 $	0.9%	7,999 $	5.5%	6,774 $	5.4%
Lab & medical supplies - Staging	114 $	0.1%	0 $	0.0%	114 $	0.1%	0 $	0.0%
Lab & medical supplies - Surveillance	2,415 $	1.7%	0 $	0.0%	2,415 $	1.7%	0 $	0.0%
Human Resources	31,074 $	22.5%	20,892 $	17.5%	31,074 $	21.5%	20,892 $	16.6%
Other supplies and materials	5,615 $	4.1%	5,045 $	4.2%	5,615 $	3.9%	5,045 $	4.0%
Fuel cost	5,719 $	4.1%	2,299 $	1.9%	5,719 $	3.9%	2,299 $	1.8%
Management	25,993 $	18.8%	23,526 $	19.7%	26,850 $	18.5%	24,384 $	19.4%
**Total Screening**	**138,245 $**	**100%**	**119,333 $**	**100%**	**144,821 $**	**100%**	**125,909 $**	**100%**
Cost per person screened	2.09 $		1.81 $		2.19 $		1.91 $	
Cost per person case detected	23,004 $		22,275 $		24,098 $		23,502 $	

Table 8 Sensitivity analysis: Population screened per year per team

Assumptions	Baseline	Parameter variation	
Population screened per year per team	66,000	72,000	54,000

	Population screened per year per team – 72,000				Population screened per year per team – 54,000			
	Traditional Team		Mini Team		Traditional Team		Mini Team	
**Capital Equipment**	**10,782 $**	**7.3%**	**8,582 $**	**6.6%**	**10,782 $**	**8.4%**	**8,582 $**	**7.8%**
**Annual Recurrent costs**	**137,225 $**	**92.7%**	**120,505 $**	**93.4%**	**117,804 $**	**91.6%**	**101,107 $**	**92.2%**
Lab & medical supplies - Screening tests (CATT & Discarded CATT)	59,185 $	40.0%	63,200 $	49.0%	44,389 $	34.5%	47,400 $	43.2%
Lab & medical supplies - Parasitological confirmation	5,607 $	3.8%	4,271 $	3.3%	4,205 $	3.3%	3,203 $	2.9%
Lab & medical supplies - Staging	124 $	0.1%	0 $	0.0%	93 $	0.1%	0 $	0.0%
Lab & medical supplies - Surveillance	2,635 $	1.8%	0 $	0.0%	1,976 $	1.5%	0 $	0.0%
Human Resources	31,074 $	21.0%	20,892 $	16.2%	31,074 $	24.2%	20,892 $	19.0%
Other supplies and materials	5,615 $	3.8%	5,045 $	3.9%	5,615 $	4.4%	5,045 $	4.6%
Fuel cost	5,719 $	3.9%	2,299 $	1.8%	5,719 $	4.4%	2,299 $	2.1%
Management	27,266 $	18.4%	24,798 $	19.2%	24,733 $	19.2%	22,268 $	20.3%
**Total Screening**	**148,006 $**	**100%**	**129,087 $**	**100%**	**128,585 $**	**100%**	**109,689 $**	**100%**
Cost per person screened	2.06 $		1.79 $		2.38 $		2.03 $	
Cost per person case detected	22,576 $		22,087 $		26,152 $		25,024 $	

Table 9 Sensitivity analysis: Specificity CATT

Assumptions	Baseline	Parameter variation	
Specificity CATT	0.948	0.993	0.835

	Specificity CATT – 0.993				Specificity CATT – 0.835			
	Traditional Team		Mini Team		Traditional Team		Mini Team	
**Capital Equipment**	**10,782 $**	**7.8%**	**8,582 $**	**7.1%**	**10,782 $**	**4.0%**	**8,582 $**	**4.5%**
**Annual Recurrent costs**	**127,935 $**	**92.2%**	**112,584 $**	**92.9%**	**259,148 $**	**96.0%**	**180,388 $**	**95.5%**
Lab & medical supplies - Screening tests (CATT & Discarded CATT)	54,253 $	39.1%	57,934 $	47.8%	54,253 $	20.1%	57,934 $	30.7%
Lab & medical supplies - Parasitological confirmation	3,479 $	2.5%	2,650 $	2.2%	80,887 $	30.0%	61,610 $	32.6%
Lab & medical supplies - Staging	114 $	0.1%	0 $	0.0%	114 $	0.0%	0 $	0.0%
Lab & medical supplies - Surveillance	1,628 $	1.2%	0 $	0.0%	38,318 $	14.2%	0 $	0.0%
Human Resources	31,074 $	22.4%	20,892 $	17.2%	31,074 $	11.5%	20,892 $	11.1%
Other supplies and materials	5,615 $	4.0%	5,045 $	4.2%	5,615 $	2.1%	5,045 $	2.7%
Fuel cost	5,719 $	4.1%	2,299 $	1.9%	5,719 $	2.1%	2,299 $	1.2%
Management	26,054 $	18.8%	23,765 $	19.6%	43,169 $	16.0%	32,609 $	17.3%
**Total Screening**	**138,717 $**	**100%**	**121,166 $**	**100%**	**269,930 $**	**100%**	**188,970 $**	**100%**
Cost per person screened	2.10 $		1.84 $		4.09 $		2.86 $	
Cost per person case detected	23,083 $		22,617 $		44,917 $		35,273 $	

Table 10 Sensitivity analysis: Serological test - SD Bioline HAT

Assumptions	Baseline	Parameter variation	
Cost serological test	0.74 $	0.60 $	
Specificity serological test	0.948	0.879	
% Discarded serological tests traditional team	8%	5%	
% Discarded serological tests mini team	15%	5%	

	Serological test – SD Bioline HAT			
	Traditional Team		Mini Team	
**Capital Equipment**	**10,782 $**	**4.9%**	**8,582 $**	**5.6%**
**Annual Recurrent costs**	**209,984 $**	**95.1%**	**144,647 $**	**94.4%**
Lab & medical supplies - Screening tests (included discarded tests)	43,272 $	19.6%	43,272 $	28.2%
Lab & medical supplies - Parasitological confirmation	59,333 $	26.9%	45,192 $	29.5%
Lab & medical supplies - Staging	114 $	0.1%	0 $	0.0%
Lab & medical supplies - Surveillance	28,101 $	12.7%	0 $	0.0%
Human Resources	31,074 $	14.1%	20,892 $	13.6%
Other supplies and materials	5,615 $	2.5%	5,045 $	3.3%
Fuel cost	5,719 $	2.6%	2,299 $	1.5%
Management	36,756 $	16.6%	27,947 $	18.2%
**Total Screening**	**220,765 $**	**100%**	**153,229 $**	**100%**
Cost per person screened	3.34 $		2.32 $	
Cost per person case detected	36,736 $		28,602 $	

Table 11 Sensitivity analysis: Serological test - HAT Sero-K-Set

Assumptions	Baseline	Parameter variation	
Cost serological test	0.74 $	0.60 $	
Specificity serological test	0.948	0.879	
% Discarded serological tests traditional team	8%	5%	
% Discarded serological tests mini team	15%	5%	

	Serological test – HAT Sero-K-Set			
	Traditional Team		Mini Team	
**Capital Equipment**	**10,782 $**	**3.3%**	**8,582 $**	**3.3%**
**Annual Recurrent costs**	**315,841 $**	**96.7%**	**252,109 $**	**96.7%**
Lab & medical supplies - Screening tests (included discarded tests)	138,211 $	42.3%	138,211 $	53.0%
Lab & medical supplies - Parasitological confirmation	57,373 $	17.6%	43,699 $	16.8%
Lab & medical supplies - Staging	114 $	0.0%	0 $	0.0%
Lab & medical supplies - Surveillance	27,172 $	8.3%	0 $	0.0%
Human Resources	31,074 $	9.5%	20,892 $	8.0%
Other supplies and materials	5,615 $	1.7%	5,045 $	1.9%
Fuel cost	5,719 $	1.8%	2,299 $	0.9%
Management	50,564 $	15.5%	41,964 $	16.1%
**Total Screening**	**326,623 $**	**100%**	**260,692 $**	**100%**
Cost per person screened	4.95 $		3.95 $	
Cost per person case detected	54,350 $		48,660 $	
